# Synthesis, characterization, and application of methylene blue functionalized reduced graphene oxide for photodynamic therapy in root canal treatment

**DOI:** 10.12669/pjms.41.2.11001

**Published:** 2025-02

**Authors:** Rizwan Jouhar, Mohamad Syahrizal Halim, Muhammad Adeel Ahmed, Faheem Shah, Sayed A. Quadri

**Affiliations:** 1 Rizwan Jouhar, Ph.D. Scholar Conservative Dentistry Unit, School of Dental Sciences, Heath Campus, Universiti Sains Malaysia, Kota Bharu 16150, Kelantan, Malaysia. Department of Restorative Dental Sciences, College of Dentistry, King Faisal University, Al-Ahsa 31982, Saudi Arabia; 2 Mohamad Syahrizal Halim Conservative Dentistry Unit, School of Dental Sciences, Heath Campus, Universiti Sains Malaysia, Kota Bharu 16150, Kelantan, Malaysia; 3 Muhammad Adeel Ahmed Department of Restorative Dental Sciences, College of Dentistry, King Faisal University, Al-Ahsa 31982, Saudi Arabia; 4 Faheem Shah Department of Chemistry, College of Science, King Faisal University, Al-Ahsa 31982, Saudi Arabia; 5 Sayed A. Quadri Division of Microbiology, Department of Biomedical Sciences, College of Medicine, King Faisal University, Al-Ahsa 31982, Saudi Arabia

**Keywords:** Endodontics, Functionalization, Graphene oxide, Methylene blue, Nanoparticles, Photodynamic therapy, Photosensitizer

## Abstract

**Background & Objective:**

Root canal infections are challenging to eradicate with conventional methods due to their complexity. Traditional chemical irrigants often fail to reach all bacterial colonies. Methylene blue (MB), used in photoactivated disinfection (PAD), generates reactive oxygen species (ROS) upon light activation, effectively killing bacteria. This study aimed to synthesize, characterize, and explore MB-functionalized reduced graphene oxide (MB/rGO) for enhanced photoactivated disinfection in root canal treatment.

**Methods:**

This in-vitro study was conducted from April 2024 to September 2024 at Universiti Sains Malaysia and King Faisal University. Graphene oxide (GO) was reduced by dispersing GO in deionized water with sonication, followed by adding sodium hydroxide (NaOH) under vigorous stirring. The suspension obtained was centrifuged, washed, and dried to yield reduced graphene oxide (rGO). For functionalization, rGO was dispersed in ethanol and mixed with methylene blue (MB) solution, followed by stirring and drying to obtain MB-functionalized rGO. The antibacterial and antifungal activities of MB alone and in combination with rGO, with or without laser exposure, were tested using the agar well diffusion method. The paired sample t-test was used to compare the inhibition zones for different treatment groups of *E. faecalis* and *C. albicans*.

**Results:**

FTIR analysis confirmed successful functionalization by identifying specific functional groups of rGO and MB. Similarly, Raman spectroscopy indicated that GO-MB had an intermediate level of defects, and SEM analysis confirmed slight morphological changes with MB molecules attached to the rGO surface. Moreover, the antimicrobial test revealed that MB/rGO with laser performed significantly better (*p*=0.042) than MB/rGO without laser and MB with laser group (*p*=0.034) against *E. faecalis*.

**Conclusions:**

The functionalization of MB with rGO and its application with laser treatment significantly enhanced antimicrobial and antifungal activity, suggesting potential benefits for endodontic treatments and other dental applications.

## INTRODUCTION

Effective endodontic disinfection is crucial for eradicating microorganisms and their by-products from the root canal system (RCS). Microorganism biofilms are the primary cause of both initial and persistent infections in the RCS.^1^ Current root canal treatment methods have not been able to decrease the disease-causing microbes to unnoticeable levels.^2^ The complex and unpredictable structure of the RCS, which contains accessory canals, isthmuses, adjacent canals, apical deltas, and recesses in oval, C-shaped, or flattened canals, hinders the thorough activity of antibacterial solutions. Consequently, innovative disinfection techniques are imperative for the effective removal of disease-causing microbes such as *Enterococcus faecalis, Bacteroides forsythus, Treponema denticola* and Yeast, mainly *Candida albicans* in RCS. Antimicrobial photodynamic therapy (aPDT) is a favorable alternate treatment that focuses on a wide range of endodontic biofilm-related infections.^3^ It employs a non-toxic photosensitizer (PS), which absorbs light from a low-intensity diode or LED light source of an appropriate wavelength, as a result of which reactive oxygen species (ROS) are produced to kill microorganisms.^4^ Methylene blue (MB) is a commonly used phenothiazinium photosensitizer in aPDT research, having been one of the first PS to obtain clinical approval.^5^

Adding Nanoparticles (NPs), ranging from 1 to 100 nm, have the potential to augment the therapeutic effectiveness of pharmaceuticals by improving the drug’s bioavailability, serum stability, and pharmacokinetics. They offer advantages by improved permeation and enable a slow, controlled discharge of active components at target places.^6^ A distinctive feature of nanoparticles is their large exterior surface area and high surface-to-volume ratio that impact their physicochemical properties. These versatile nanoparticles are used in irrigants, obturating materials, and intracanal medicaments, among other components of endodontic therapy.^7^ Pagonis et al. found that poly (lactic) co-glycolic acid nanoparticles (PLGA NPs) loaded with MB are effective against E. faecalis, a prevalent bacteria in endodontic infections.^8^

One of the commonly used nanoparticles is graphene oxide.^7^,^9^ Graphene is a two-dimensional single layer of carbon atoms organized in a honeycomb framework, which has garnered significant attention from researchers due to its remarkable antibacterial properties. It is typically linked to multiple mechanisms of action, such as oxidative stress, phospholipid molecule withdrawal, bacterial cell membrane breakdown and entrapment, and self-killing effect.^9^ Graphene oxide (GO), serves as an excellent carrier for medications and biomolecules, improving the mechanism of action and biological activity of biomaterials to enhance their antimicrobial activities.^10^

Studies have shown that contemporary root canal disinfection methods are inadequate in ensuring complete bacterial eradication, leading to high failure rates and persistent infections.^2^-^3^ Contemporary root canal disinfection methods are inadequate in ensuring complete bacterial eradication, leading to high failure rates and persistent infections. The application of MB/rGO NPs for photoactivated disinfection offers the potential to improve root canal treatment outcomes. Therefore, this study aimed to synthesize, characterize, and explore the application of MB functionalized rGO (MB/rGO) for photoactivated disinfection in root canal treatment.

## METHODS

This in-vitro study was conducted from April 2024 to September 2024.

### Ethical Approval:

It got approval from University Sains Malaysia (USM/JEPeM/KK/24030230, dated June 13, 2024) and King Faisal University (KFU-REC-2024-MAR-ETHICS2072, dated March 20, 2024).

### Reduction of Graphene Oxide:

To reduce graphene oxide (GO), commercially available GO with 99% purity and particle size range between 30 to 50 nm was used. First, 100 mg of GO was dispersed in 500 ml of deionized water using vigorous sonication for two hours to produce a homogeneous solution. From this, 250 ml of the suspension was moved to a 500 ml beaker, and 2-3 ml of 8 M NaOH solution was added dropwise under vigorous stirring at 1000-1200 rpm for five hours, resulting in a black suspension. The mixture was subjected to centrifugation at 10,000 rpm for 30 minutes. The resulting precipitate was washed three times with deionized water and ethanol, using 2 mL of 0.5 M hydrochloric acid (HCl) in each wash to adjust the pH from 12 to 7.5. The final product was dried at 80°C for 8 hours, producing reduced graphene oxide (rGO) powder.^11^

### Functionalization of Methylene Blue with Graphene Oxide:

For functionalization, 50 mg of rGO was dispersed in 50 ml ethanol via sonication for one hour, and 50 mg of methylene blue (MB) was dissolved in 100 ml ethanol. The MB solution was added dropwise to the rGO suspension under mild stirring at 40-45°C for 6 hours. The resulting MB/rGO slurry was dried at 45°C to obtain functionalized Mb/rGO powder.

### Antibacterial and Antifungal Activity Testing:

The agar well diffusion method was used and repeated three times. Each time testing was done under similar conditions on Mueller-Hinton Agar for *E. faecalis* and *C. albicans*.^12^ *Enterococcus faecalis* ATCC 29212 and *Candida albicans* ATCC 90028 were tested. A colony suspension was prepared by transferring 2-3 colonies from a fresh overnight culture plate, into sterile physiological saline. The suspension was vortexed, and the turbidity was adjusted to 0.5 McFarland and spread on Mueller-Hinton agar plates. The test compounds were blinded as, A: MB alone, B: MB/rGO, AL: MB with laser, BL: MB/rGO with laser. Two wells of 6 mm were cut into the agar, and 100 µL of test compounds were added. AL and BL compounds were exposed to a 665nm laser light (F3WW, Foshan, Guangdong, China) at 200 mW power for two minutes after the placement of PS in the well. Plates were incubated at 37°C for 24 hours, and inhibition zones were noted as an area of no growth and measured by a scale as the diameter, from edge-to-edge of the clear zone, in millimeters. A purity plate and sterility plate were included as controls.

### Statistical analysis:

The data was analyzed using SPSS version 23.0. The zones of inhibition for different treatment groups were reported as means and standard deviations. The paired sample t-test was used to compare the inhibition zones for different treatment groups of *E. faecalis* and *C. albicans*. A *p*-value of less than 0.05 was considered statistically significant.

## RESULTS

### FTIR (Fourier-Transform Infrared Spectroscopy):

**Fig.1:**
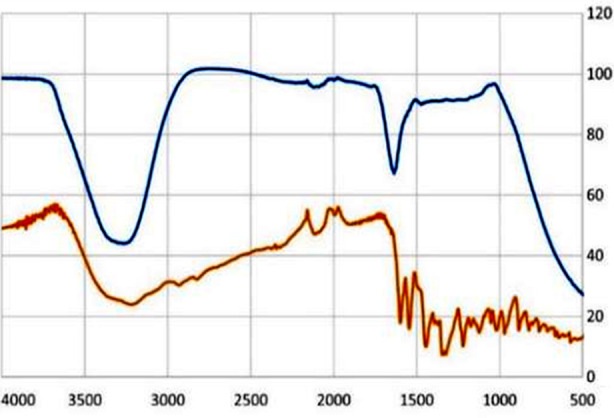
Spectrum in blue: rGO, Spectrum in brown: MB/rGO FTIR (Fourier-Transform Infrared Spectroscopy) spectral analysis.

FTIR analysis was performed on rGO and MB/rGO samples, as shown in Fig.1. The rGO spectrum displayed characteristic peaks at 3750–3000 cm^−1^ and 1640 cm^−1^, indicating oxygen-containing functional groups (hydroxyl, carbonyl, and epoxy) typical of rGO. In MB/rGO, bands of MB appeared in the 1600–600 cm^−1^ region. A shift from 1640 cm^−1^ to 1598 cm^−1^ and a reduction in intensity were observed, along with stretching vibrations at 1598, 1545, and 1220 cm^−1^, corresponding to C=N^+^ (CH_3_)_2_, C—N, and C—C bonds of MB on GO. Peaks at 1491 and 1355 cm^−1^ indicated C=S^+^ stretching, while C—H vibrations in N(CH_3_)_2_, appeared at 1444 and 1392 cm^−1^. Additional peaks in the MB/rGO spectrum confirmed the successful adsorption of MB onto rGO, indicating successful functionalization.^13^

### Raman Spectroscopy:

**Fig.2:**
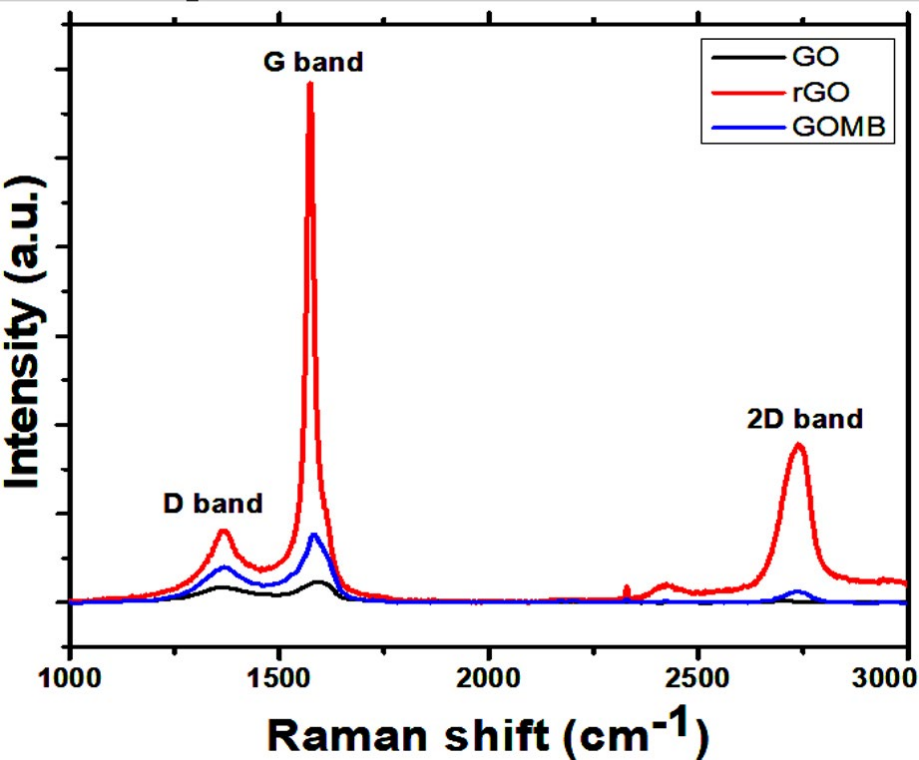
Room temperature Raman spectrum of GO, rGO, and MB/rGO.

Raman spectroscopy was performed at room temperature to analyze the internal structure, disorder, and crystallinity of GO, rGO, and MB/rGO samples. Fig.2 shows the spectra in the range of 1000–3000 cm^−1^, highlighting the D, G, and 2D bands characteristic of carbon materials. The D band indicates structural disorder from sp² bonds, while the G band reflects pristine carbon atoms in GO or rGO. The D/G intensity ratio (I_D_/I_G_) reveals defect levels: 0.81 for GO, 0.144 for rGO, and 0.55 for MB/rGO. This suggests that rGO has fewer defects, while GO and MB/rGO show some degree of disorder.^14^

### Scanning Electron Microscopy (SEM) Analysis:

**Fig.3:**
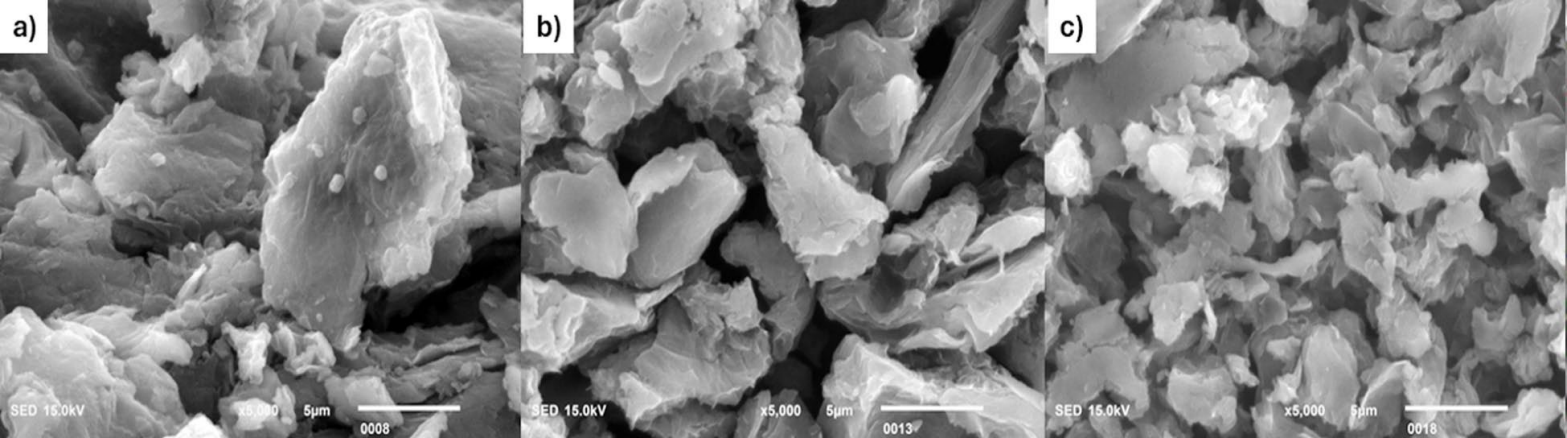
SEM images of a) GO, b) rGO, and c) MB/rGO.

SEM was used to examine the morphology of GO, rGO, and MB/rGO. The GO image (Fig.3a) shows thick, flaky sheets with wavy edges and pores, featuring oxygen-containing groups that increase interlayer spacing and create wrinkles.^15^ After reduction to rGO (Fig.3b), the surface becomes rough, preventing tight stacking of layers, with a clean, impurity-free appearance.^11^ In Fig.3c, MB molecules are seen adhering to the rGO surface, confirming successful adsorption with minimal change in morphology.

### Antibacterial and Antifungal Activity:

**Fig.4:**
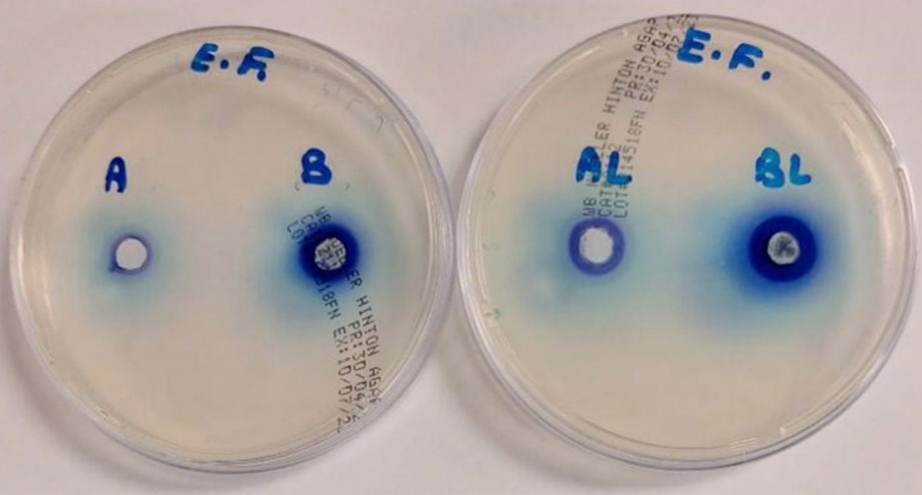
Well diffusion test of E. faecalis.

**Fig.5:**
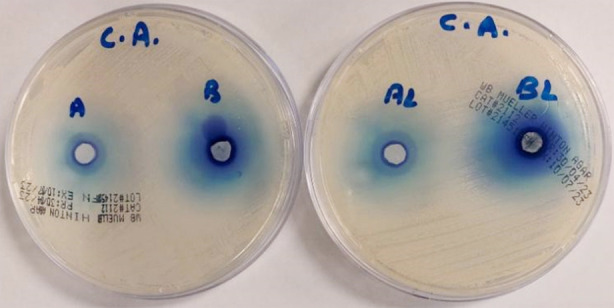
Well diffusion test of C. albicans.

The antibacterial and antifungal activity of methylene blue (MB) and MB/rGO with and without laser exposure was evaluated against *Enterococcus faecalis* and *Candida albicans*. For *E*. *faecalis*, the highest inhibition zone (19.66 ± 1.15 mm) was observed for BL (MB/rGO with Laser) whereas the lowest inhibition zone (10.00 ± 2.00 mm) was noticed for A (MB). While comparing the groups, BL performed significantly better (*p*=0.042) than B (MB/rGO) and AL (MB with Laser) group (*p*=0.034). For *C. albicans***,** BL showed the highest antifungal activity (34.66 ± 0.57) and A (MB) showed the least inhibition zone of 25.00 ± 1.00 mm. When the groups were compared, BL performed significantly better than the AL group (p=0.039). Similarly, the B group (29.33 **±** 2.08) performed significantly better (p=0.023) than the A group (25.00 ± 1.00) as shown in Tables-I and II. Overall, the combination of MB/rGO with laser (BL) proved to be the most effective treatment with highest antimicrobial activity against both E. faecalis and C. albicans (Fig.4 and 5).

**Table-I:** Zone of inhibition.

Antimicrobial and Antifungal activity		Zone of inhibition in mm Mean ± SD	p-value
***E. faecalis***	A (MB)	10.00 ± 2.00	0.184
	AL (MB + Laser)	12.00 ± 2.64	
	B (MB/rGO)	14.00 ± 1.00	0.042
	BL (MB/rGO + Laser)	19.66 ± 1.15	
***C. albicans***	A (MB)	25.00 ± 1.00	0.067
	AL (MB + Laser)	30.33 ± 1.52	
	B (MB/rGO)	29.33 ± 2.08	0.057
	BL (MB/rGO + Laser)	34.66 ± 0.57	

**Table-II:** Comparison of the zones of inhibition for different treatment groups of E. faecalis and C. albicans.

Antimicrobial and Antifungal activity		Zone of inhibition in mm Mean ± SD	p-value
***E. faecalis***	A (MB)	10.00 ± 2.00	0.147
	B (MB/rGO)	14.00 ± 1.00	
	AL (MB + Laser)	12.00 ± 2.64	0.034
	BL (MB/rGO + Laser)	19.66 ± 1.15	
***C. albicans***	A (MB)	25.00 ± 1.00	0.023
	B (MB/rGO)	29.33 ± 2.08	
	AL (MB + Laser)	30.33 ± 1.52	0.039
	BL (MB/rGO + Laser)	34.66 ± 0.57	

## DISCUSSION

The study focused on synthesizing, characterizing, and evaluating the application of methylene blue (MB) functionalized reduced graphene oxide (MB/rGO) for photoactivated disinfection in root canal treatment. The results demonstrated successful functionalization, as evidenced by the identification of specific functional groups of rGO and MB. Furthermore, antimicrobial testing revealed that MB/rGO combined with laser treatment was significantly more effective (p=0.042) than MB/rGO without laser and the MB with laser group (p=0.034) in combating E. faecalis.

The introduction of NPs in dentistry, mostly in endodontics, shows significant potential for improving treatment outcomes. Traditional mechanical instrumentation and irrigation often fail to cover all sites, leaving behind remaining biofilms and bacteria that can undermine treatment success.^16^ Recently, the use of graphene-based nanomaterials (GBNPs) loaded with a photosensitizer has shown several benefits. When exposed to a specific wavelength of light, these materials boost the creation of ROS, resulting in a more potent bactericidal effect from the nanoparticles. Therefore, this study investigates the potential of MB functionalized with reduced graphene oxide (rGONPs) to functionalize MB, aiming to enhance its effectiveness for root canal disinfection. GBNPs have proven highly efficient in preventing bacterial attachment on various dental surfaces, such as root canal walls and dental implants.^8^ In the present study, rGONPs incorporated MB photosensitizer has shown encouraging outcomes in enhancing endodontic disinfection.

Raman spectroscopy proves highly effective in examining defects and disorders within crystal structures. The present study reveals an I_D_/I_G_ ratio of 0.81 for GO, 0.144 for rGO, and 0.55 for MB/rGO, indicating successful reduction and functionalization. These results align with prior findings in which a slight decrease in the I_D_/I_G_ ratio from 0.94 in pristine samples to values of 0.88, 0.86, 0.80, and 0.76 as the reduction temperature increases to 125, 150, 175, and 200 °C, respectively.^17^ This decrease denotes the removal of the majority of oxygen-containing functions, which causes the sp^2^ carbon domains to increase.^18^ Additionally, the Raman spectra show a less pronounced band at about 2700 cm^1^, which may shed light on the number of layers within GO sheets.^19^

FTIR spectroscopy also validated the elimination of these oxygen functional groups to create rGO through thermal reduction. GO’s spectrum showed characteristic peaks at 3750–3000 cm^−1^ and 1640 cm^−1^, indicating hydroxyl, carbonyl, and epoxy groups, which were less pronounced in rGO. Shifts in MB/rGO spectra confirmed the successful adsorption of MB onto rGO, particularly through peaks at 1598, 1545, and 1220 cm^−1^ related to C=N^+^(CH_3_)_2_, C—N, and C—C bonds. These results are consistent with studies showing flattened peaks at 3395 cm^−1^ (hydroxyl) and 1721 cm^−1^ (carbonyl) due to the reduction process.^17^,^20^

SEM analysis revealed that GO has a complex, flaky, and porous structure with wavy edges, consistent with previous studies.^21^ In contrast, rGO exhibits a more wrinkled and grouped structure due to the heat reduction process. Both GO and rGO show no charging effects in SEM imaging, indicating their electrically conductive nature.^22^

The present study supported that MB, both alone and in combination with rGO, exhibits antibacterial and antifungal activities against *E. faecalis* and *C. albicans* which are known for their resistance in endodontic treatments. However, the efficacy of these treatments is further enhanced when combined with laser exposure. The zones of inhibition for *C. albicans* were highest when MB/rGO was exposed to laser light (34.66 ± 0.57) in comparison to MB alone (25.00 ± 1.00). These results are consistent with another study, which showed that MB alone exhibited an antifungal effect on *C. albicans* following photoactivated disinfection. However, silver nanoparticles showed an increase in antifungal activity against all species of Candida.^23^

Another study found that graphene oxide-double antibiotic paste (GO-DAP) was the only substance that was able to both drastically lower and eliminate the *E. faecalis* bacterial load in a single day, showing a statistically higher reduction in bacterial count compared to DAP.^24^ According to Ioannidis *et al.*, Ag-GO reduced the bacterial load by 57%, which was less effective than the standard endodontic irrigant, NaOCl, available in the market.^25^ Conversely, Sharma et al. found that the total microbial biovolume of Ag-GO nanoparticles was 86.85%, slightly greater than NaOCl (80.40%), although this difference was not statistically significant.^26^ These findings underline the use of MB and rGO in PAD to improve antimicrobial effectiveness against resistant pathogens like *E. faecalis* and *C. albicans*.

The study’s findings suggest potential for improved root canal disinfection outcomes using methylene blue and reduced graphene oxide nanoparticles with laser exposure. This approach may increase treatment success rates, reduce post-treatment infections, and offer an alternative to traditional irrigants. Further research is necessary to optimize dosing, explore combination therapies, and establish clinical protocols. This innovation holds promise for managing antibiotic-resistant endodontic infections.

### Limitations:

First, only two microbial strains (E. faecalis and C. albicans) were tested, which may not represent the broader antimicrobial spectrum. Second, the well-diffusion test performed may not accurately represent the antimicrobial efficacy in a real-world scenario. Further studies are desirable to include more microbial strains and biofilms.

## CONCLUSION

The functionalization of MB with rGO and its application with laser light treatment significantly enhanced antimicrobial and antifungal activity, suggesting potential benefits for endodontic treatments and other dental applications. Additionally, FTIR, RAMAN, and SEM analysis verified the existence of functional groups specific to graphene oxide and methylene blue, indicating successful functionalization.

### Author’s Contribution:

**RJ:** Conceived and designed the study, Literature review, Data acquisition, Data analysis, and Drafted Manuscript. **MSH:** Designed the study, Interpretation of results, Critical review of the manuscript, and Supervision. **MAA: I**nterpretation of results, Critical review of manuscript, and Supervision. **FS:** Data acquisition, Interpretation of results, Critical review of manuscript. **SAQ:** Data acquisition, Interpretation of results, Critical review of manuscript. All authors have read and approved the final manuscript and are responsible for the integrity of the research.
